# A Detailed Properties Comparison of an Automotive Sealant Nozzle Produced Using Three Metal Additive Manufacturing Technologies

**DOI:** 10.3390/ma17153637

**Published:** 2024-07-23

**Authors:** Jaime Ortiz-Cañavate, Santiago Ferrandiz, Carlos A. Bloem, Javier Igual, Jose Ramon Blasco

**Affiliations:** 1 Manufacturing Strategy & Advanced Planning, Lean Manufacturing Europe, Ford España S.L., Polígono de Almussafes, 46440 Valencia, Spain; 2 Technology and Materials Engineering Institute, Universitat Politècnica de València, 03801 Alicante, Spain; sferrand@mcm.upv.es; 3 New Manufacturing Processes Department, AIDIMME, Av. Leonardo Da Vinci, 38 Paterna, 46980 Valencia, Spain; cbloem@aidimme.es (C.A.B.); jrblasco@aidimme.es (J.R.B.); 4 Innovation and New Technolgies Department, Ford España S.L., Polígono de Almussafes, 46440 Valencia, Spain; jigualc1@ford.com

**Keywords:** additive manufacturing, 3D printing, metal, automotive, industry, nozzle

## Abstract

Choosing the right metal AM equipment and material is a highly intricate process that forms a crucial part of every manufacturing company’s strategic plan. This study undertakes a comprehensive comparison of the performance and material properties of three Metal Additive Manufacturing (AM) technologies: Powder Bed Fusion (PBF), Metal Filament Deposition Modeling (MFDM), and Bound Metal Deposition (BMD). An automotive nozzle was selected and manufactured using all three technologies and three metallic materials to understand their respective advantages and disadvantages. The samples were then subjected to a series of tests and evaluations, including dimensional accuracy, mechanical properties, microstructure, defects, manufacturability, and cost efficiency. The nozzle combinations were PBF in aluminum, MFDM in stainless steel, and BMD in hard tool steel. The results underscore significant differences in functionality, material characteristics, product quality, lead time, and cost efficiency, all of which are crucial factors in making equipment investment decisions. The conclusions drawn in this paper aim to assist automotive industry equipment experts in making informed decisions about the technology and materials to use for parts with characteristics like these. Future studies will delve into other technologies, automotive components, and materials to further enhance our understanding of the application of metal AM in manufacturing.

## 1. Introduction

The principle of AM, also called 3D printing [1], consists of adding layers of material on top of each other to build the final component. This part-making method fundamentally differs from traditional subtractive processes (ISO/ASTM International 2015). The main competitive benefits are cost efficiency, short lead times, and environmental sustainability. Research and development in AM started in the aerospace, medicine, transportation, energy, and consumer products industries. Recently, the automotive industry has integrated AM into its daily operations as a key source of components [2]. It is consolidated as a key manufacturing process for its competitive costs, reduced lead times, and design flexibility. Traditionally, AM production has been very active in using polymers with four main process families. These range from the most used Filament Deposit Modeling (FDM), through Stereolithography (SLA) for accurate, watertight, and durable parts; Continuous Filament Fabrication (CFF) for fiber-reinforced materials; and MultiJet Fusion (MJF) for high productivity.

However, for applications that require demanding mechanical characteristics, metal AM is necessary. The five metal AM technologies are Powder Bed Fusion (PBF), Directed Energy Deposition (DED), Metal Fused Deposition Modeling (MFDM) process, Bound Metal Deposition (BMD), and the recently fast-growing Metal Binder Jetting (MBJ). The most common metals used are stainless steel, titanium, hard steel, copper, and aluminum. Additionally, the number of metal AM technologies and Original Equipment Manufacturers (OEMs) competing in the industry is very high. For each of these families, numerous OEMs are launching products to the market, making purchasing decisions extremely complex. The main dilemma that design and manufacturing engineers face is where polymer AM does not provide the required properties: “What metal AM technology and which material are the right ones to achieve the highest quality, cost, and lead time results?” [3].

Numerous authors describe AM applications in the automotive industry and mention specific applications, advantages, and opportunities. In this area, several researchers highlight examples of different metal AM solutions, materials, and processes applied in the industry to produce different types of components. An example is Vasco [4], who documented work carried out in the automotive industry with AM, including application examples and the advantages. Also, Asnafi [5] compiled in 11 papers numerous metal AM solutions for tooling manufacturing. In this line of research, Leal [6] describes experimentation on stamping die inserts manufactured in metal AM. Furthermore, organizations like NASA are developing standards for quality control of parts manufactured using AM [7].

Machine spares, equipment tooling, new product prototypes, and serial manufacturing parts are the most indicated applications to produce with AM in the automotive manufacturing industry [8,9]. To date, AM technologies, mainly with polymers, have demonstrated numerous applications with average savings of 75%, production times of days, and significant weight reduction of up to 95% with a significant impact on environmental sustainability [10]. Nevertheless, whenever metal AM is required for functional requirements, there are multiple options, and a wealth of complex vendor technical information needs to be considered when pursuing equipment purchases aiming to maximize performance, reduce costs, and ensure competitive timing [11].

This research describes the leading metal AM technology families available and their advantages and disadvantages. Next, three combinations of metal AM technologies and materials are presented, PBF with EN AW 4343 aluminum, MFDM with ASI 316L stainless steel, and BMD with 17-4PH type ASI 630 hard tool steel, to produce a sealant nozzle used in a vehicle manufacturing plant. These combinations were selected following intuitive steps to achieve maximum efficiency: lowest investment first, reduced cost second, and shorter lead time third. Consequently, this paper compares these samples’ functional performance, dimensional accuracy, mechanical properties, microstructure, general manufacturing quality and defects, and cost savings. Finally, considering the experimental results and vendor technical information, this paper recommends the best technology suited for this application.

This work aims to provide additional information on metal AM manufacturing of small automotive parts to support a decision-making model for equipment acquisition.

## 2. Description and Comparison of Metal Additive Manufacturing Technologies 

**Figure 1:**
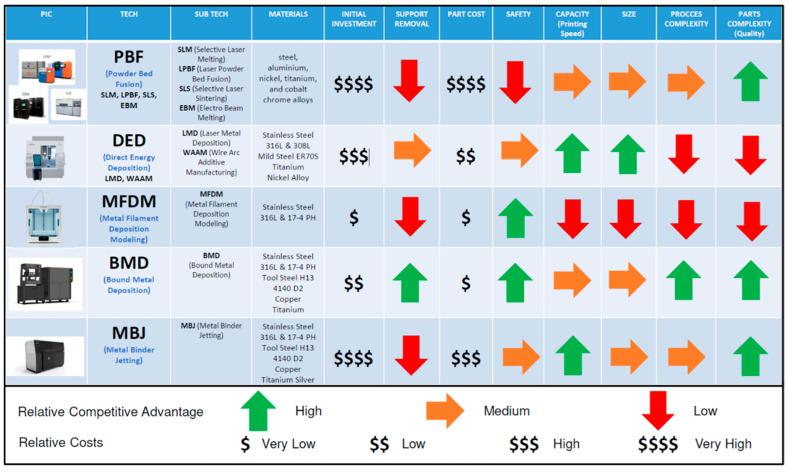
Metal 3D printing technologies comparison—chart courtesy of Ford España S.L.

According to I. Gibson [12], the main metal additive manufacturing technologies are PBF [13], DED, MFDM [14], BMD, and MBJ [15] (Table 1). Figure 1 compares the leading metal AM technologies, highlighting each competitive advantage area. This study selected PBF, MFDM, and BMD from the list of families as the relatively most mature technologies offering equipment that can be easily installed in automotive manufacturing facilities and meet quality and cost requirements.

## 3. Material and Methods

**Figure 2:**
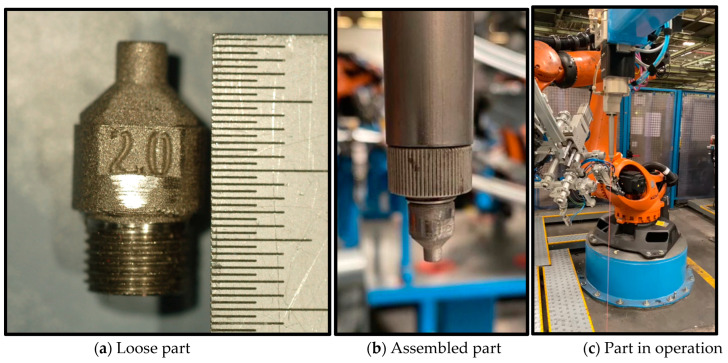
(**a**–**c**) Pictures of the sealant nozzle included in this paper—courtesy of Ford España S.L. [16].

Figure 2 shows a 2 mm diameter sealant nozzle and two pictures of its application in four robotic applications of the clinching cells for body closures in the Ford Valencia Stamping and Body Plant. IBETAMATE (TM) 210G Structural Adhesive is the product applied to seal gaps in sheet metal preassemblies to ensure that these parts are watertight after operation. These nozzles, assembled at the end of a robot, operate with pressure tolerances of 5 to 170 bar and typically an average of 50 bar. Spares produced with polymers on FDM, SLA, or MJF could not withstand these high working pressures. The thread on these plastic parts was not sufficiently robust and failed functional validation on production equipment.

A nozzle for distributing sealant onto automotive body panels was selected for these trials; its actual application is illustrated in Figure 2. The experiments included functional validation of the actual production equipment, microscopic analysis of part sections, material analyses, and mechanical tests. Additionally, the main differences in functionality, mechanical properties, microstructure, and general manufacturing quality in specific areas are discussed in this paper.

**Figure 3:**
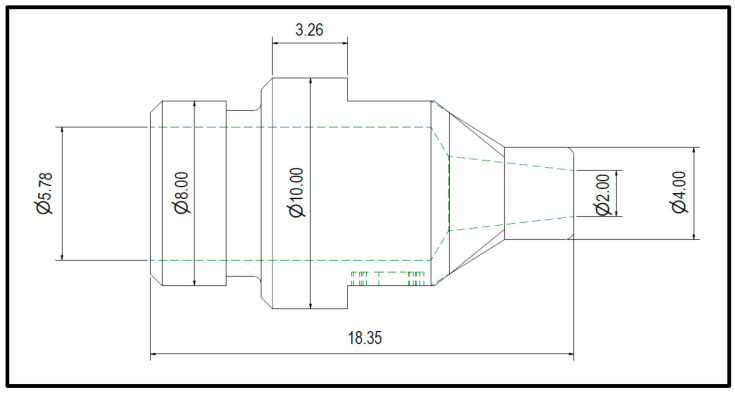
CAD drawing of the nozzle with dimensions (mm).

**Figure 4:**
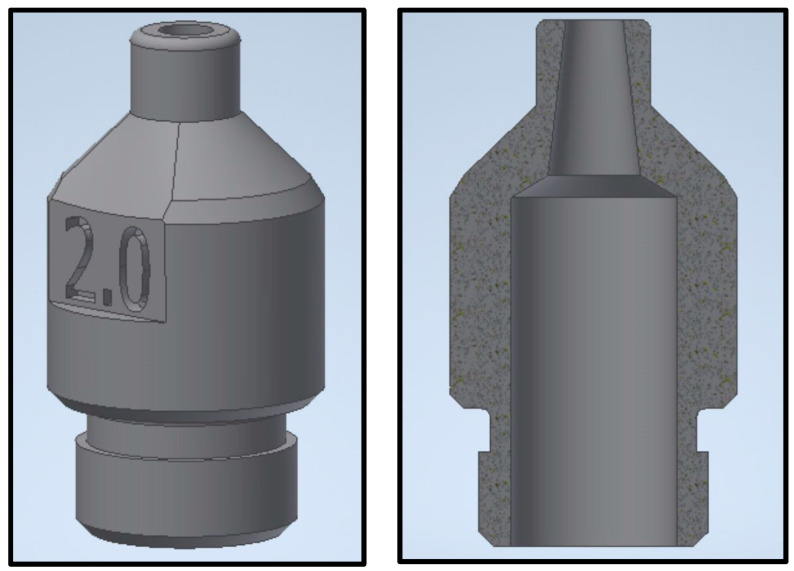
3D model and section.

Consequently, five nozzles using the same 3D model were produced with sealer exit facing down in this order on PBF, MFDM, and BMD facilities using a 3D model and dimensions shown in Figure 3 and Figure 4. From each batch, one part was trialed on the actual production cell to identify any functionality issues. The other samples were sectioned for material composition, hardness, yield strength, defects, microstructure, and porosity analyses.

Below are the metal AM production facilities used in this study:

- MFDM Ultimaker S3 with BASF 316L filament (located at Ford Almussafes 3D Printing Lab, Almussafes, Spain).
- Desktop Metal Studio 2 BMD AM System including printers and furnace (also located at the Ford Almussafes 3D Printing Lab).
- LPBF PRIMA Print Genius printer, with a laser fusion source (located at AIDIMME Institute in Paterna–Valencia, Spain).

Table 2 shows the equipment and software used to produce the samples it includes the main parameters and settings applied to each printer. 

Additionally, the following software applications were essential to the work of this study:

- Inventor 2024.1 (Autodesk, Inc., San Rafael, CA, USA) and Solidworks 2023 SP3.0 (Dassault Systèmes Solidworks Corp., Waltham, MA, USA) for 3D model creation and optimization.
- Magics 26.01 (Materialise NV, Leuven, Belgium) software for printing job preparation.
- 3YourMind 23.11.2 (3YOURMIND GmbH, Berlin, Germany) to manage printers and jobs and estimate costs.
- PowerApp designed by the Ford team to coordinate the complete process flow from concept to documentation.
- AZTEC (v6.0) Material Composition Analysis Software
- Buehler Omnimet 9.9.3.1 Metallurgical Microscope Software.

**Figure 5:**
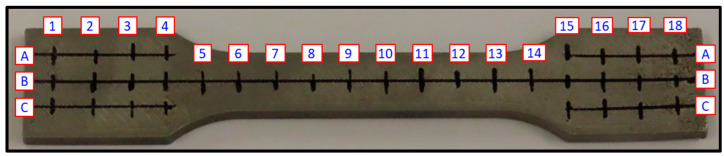
Hardness measurement markings on the yield strength specimen using numbers from 1 to 18 for columns and letters A to C for rows to locate exact position of individual hardness measurements.

The test equipment used in this work was an Olympus BX 51M metallographic microscope and a Stereo Macroscope Zeiss Discovery V-8. The main characteristics analyzed were general appearance, external surface, hardness, internal structure, and visual defects. Yield strength data were acquired with Ibertest equipment at Universitat Politècnica de València Alcoi Campus facilities using standard specimen UNE-EN ISO 6892-1 [17]. Testor hardness measurement equipment from Company Instron Wolpert located in Ford España S.L. facilities was used. Vickers hardness test (ISO 6507 [18]) was applied to all specimens in a replicated pattern, as shown in Figure 5. We used a Surface Electronic Microscope (SEM) from JEOL JSM-5310 with an Oxford Instruments Xplore 30 X-ray beam and AZTEC Material Composition Analysis Software.

Part porosity was measured using an open-source image processing software package, Fiji, popular open-source software. ImageJ (https://imagej.net/ij/, accessed on 1 June 2024) focuses mainly on biological-image analysis but can be used for metal porosity [19].

## 4. Main Results

The tests provided data on function and dimensions, material composition, mechanical properties, metallurgical characterization, quality defects including porosity, and cost comparison. In the next section, the results of these tests are described, and the three technologies and materials are compared.

### 4.1. Functional and Dimensional Results

**Figure 6:**
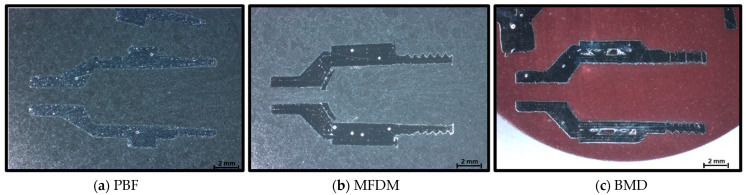
(**a**–**c**) Sample part sections (×10). Note that dimensional differences in the parts result from small variations in the sectioning process.

Nozzle sections of the samples manufactured in this study are shown in Figure 6 [16].

From a functional point of view, the part in Figure 6 produced in aluminum failed functional trials because the thread did not withstand the axial forces resulting from the operational pressure. However, the parts in Figure 6b,c manufactured in stainless steel ASI 316L and hard tool steel 17-4PH type ASI 630 could operate without issues. As with plastic and resin, aluminum was not suitable for the application.

At first glance, the sections in Figure 6a–c show a significant difference immediately; BMD does not print solid parts. During part sectioning, it was found that the BMD manufacturing process automatically incorporates an internal lattice structure. A lattice structure consists of repeating patterns that, when connected, form three-dimensional shapes, resulting in a non-solid internal volume. According to the machine manufacturer, this lattice is created using the standard settings of the slicing software to allow for better binder extraction in the oven. Consequently, it also results in a reduced total weight. These sections highlight another evident result: the PBF process produces parts with a smoother external surface than MFDM and BMD. And finally, MFDM sections immediately manifest in an internal material detachment issue.

While the PBF process produces parts in final shape and size, part supports need to be included to avoid unintended part distortions. Even though designers minimize the use of supports, these features are critical and significantly influence the final shape outcome. In this case, supports did not have a negative effect.

The MFDM process requires printing with oversized dimensions, and later, the sintering process, executed at the filament manufacturer location, brings the component to the final size. These oversized dimensions needed to be calculated by hand and incorporated into a modified .stl model for printing. Time, experience, and several iterations were necessary to achieve the required level of accuracy.

BMD also prints parts with oversized dimensions using a software algorithm that ensures very accurate final size and shape. The logic incorporates an internal lattice to facilitate binder extraction and thus lighten the parts while maintaining the required mechanical properties and external shape.

**Figure 7:**
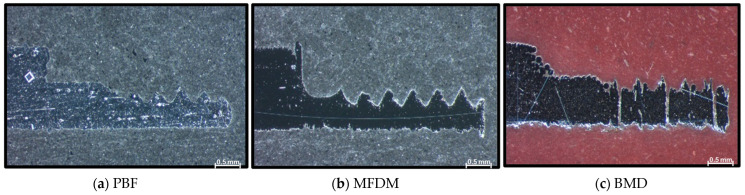
(**a**–**c**) Sections (×32) of parts showing the thread area; note that the MFDM sample has already been reworked with a threading tool.

Figure 7a–c highlight the key differences among the three samples in the threaded area. Parts manufactured in PBF, provide a solid, rounded, but somewhat inaccurate shape that failed normal operation even after reworking with a threading tool. On the other hand, the MFDM part, as shown in Figure 7b, initially showed a very irregular shape; however, after post-processing, the thread provided sufficient resistance to withstand process pressures. Finally, the BMD part in Figure 7c showed an unexpected defect pattern in the threaded area, a series of parallel lines lacking material at regular distances. However, these defects did not impact functionality, and the part was validated on production equipment successfully. This condition was not present when the threaded area was printed with an oversized cylindrical shape and consequently machined to the final shape with a threading tool. 

**Figure 8:**
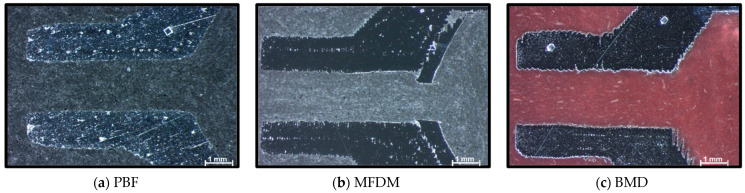
(**a**–**c**) Sections (×32) of parts showing the sealant outlet area.

Pictures in Figure 8 show significant differences in the outcomes from each AM process in the sealant outlet area. The PBF parts shown in Figure 8a highlight a smooth and wavy external surface, while MFDM in Figure 8b and BMD in Figure 8c produce shapes with high cylindrical accuracy. However, the MFDM sample in Figure 8b shows obvious material detachment around the whole internal area. Nevertheless, these evident internal defects did not interfere with sealant flow, and the parts were functionally sufficiently good for the application. BMD parts (Figure 8c) showed very good dimensional capabilities. However, long and thin superficial peaks were present, mainly on one side of the internal area. According to the OEM, this defect on one side of the part results from melted raw material flow during printing, which is explained by gravity influence depending on part orientation. In this case, as with MFDM, these defects did not impact the flow of sealant in BMD parts.

**Figure 9:**
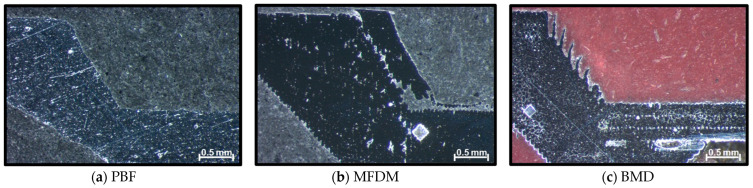
(**a**–**c**) Sections (×50) of parts showing the inner side of the section reduction area.

In the area displayed in Figure 9, as seen in Figure 8, PBF parts (Figure 9a) showed a compact structure with smooth external contour. The MFDM samples (Figure 9b) highlight a big detachment irregularity on the inner side. The BMD sections (Figure 9c) display areas with an internal lack of material and thin peaks in the internal surface. 

MFDM and BMD defects result from the nature of the AM processes, where material with binder is deposited layer over layer, and this was evident in the entire sectioning process. Nevertheless, the end result of these two processes is different and, therefore, could affect functionality depending on the application. Part orientation, printing temperature, and speed are the main factors that influence the creation of these defects.

**Figure 10:**
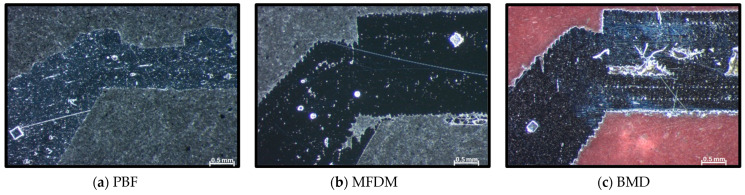
(**a**–**c**) Sections (×50) of parts show the narrowing area’s outer section to the sealant outlet area.

Again, Figure 10 shows defects similar to those described in previous figures. However, the section in Figure 10, made with PBF, depicts a much more irregular external surface than the other sections, probably the result of the abrupt diameter change. Interestingly, on this side of the nozzle, the BMD part in Figure 10c does not produce the tall and thin peaks as on the opposite side. However, small areas with no material resulting from the lattice structure are evident.

### 4.2. Material Composition

Table 3 summarizes the values from material spectrographs of the three samples, providing detailed element composition for the three materials: an aluminum alloy used in PBF, stainless steel in MFDM, and hard tool steel in BMD. As expected, when comparing the ferrous materials, 17-4PH ASI 630 shows a higher percentage of Fe, Cr, and Si, while ASI 316L includes a higher amount of Ni and Mo.

### 4.3. Mechanical Properties

Table 4 reveals yield strength and hardness measured on samples manufactured with the sample processes and materials. According to data provided by the AIDIMME institute on samples manufactured recently, PBF parts fabricated in aluminum resulted in a yield strength of 232 MPa. Hardness measurements on the physical part resulted in 109 HV10. As a comparison, previously made hardness tests on samples made with PBF and 17-4PH resulted in 747 Mpa, which is comparable with samples made with BDM.

On the other hand, according to the BASF catalog, MFDM parts manufactured with stainless steel 316L would provide up to 680 Mpa yield strength and 257 HV10 hardness. However, hardness measurements conducted in this work resulted in 128 HV10, half of the hardness specified by the filament supplier, but only 16% lower values than traditional manufacturing processes. These significant differences might result from process variability between experiments in the lab and actual production. They highlight the potential uncompetitive position of MFDM compared with PBF for the same material. Also, 316L stainless steel showed high mechanical property potential when using AM [20].

As depicted in Table 4, BMD parts made with 17-4PH hard tool steel and internal lattice showed a yield strength of 724 Mpa. As stated by the vendor, this value is slightly higher than that of stainless steel solid parts manufactured with MFDM. Also, the BMD process produced samples with a hardness of 307 HV10, nearly three times the hardness measured on the other two process samples. Note that hardness measurement using Rockwell C (HRc) was not applicable on parts manufactured with an internal lattice or softer materials. Therefore, Vickers Hardness HV10 was used to cover the whole range of metallic materials in line with metal AM OEM catalogs and other publications [21].

A comparison of these measurements with traditionally produced alloys provides insight into the impact of AM processes on mechanical properties [22]. For example, yield strength values of traditional alloys are similar for Al 4343 and 316L. However, 17-4PH AM parts tests provided 25% lower values; the most probable reason is the internal lattice structure present in the BMD process. Furthermore, the hardness values of Al 4343 PBF-produced parts are 25% higher than traditional alloys’ maximum values. On the other hand, 316L MFDM and 17-4PH BMD samples show lower hardness values of 16% and 15%, respectively, compared with non-AM production parts.

### 4.4. Metallurgical Characterization and Porosity Defects

**Figure 11:**
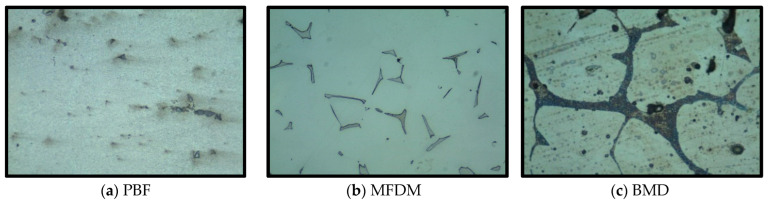
(**a**–**c**) Sections (×500) of parts showing the metallurgical structure.

The nozzle printed with PBF in aluminum, Figure 11a, showed a very consistent and homogeneous eutectic structure with a certain level of microporosity (black areas), as shown in Table 5, measured at 1.026% with an average particle size of 8.398 µm. The presence of no significant metallographic defects was in line with the results of other authors [23]. This internal structure is significantly different from the typical one present in aluminum casting parts, i.e., automotive engine aluminum machined parts [24].

Parts fabricated with MFDM in 316L stainless steel, Figure 11b, showed dispersed ferritic islands (5% in volume) and grain-boundary carbide particles in an austenitic matrix with a very low porosity, resulting in 0.414% and 2.655 µm average size (Table 5). Again, the sections did not show significant defects.

The BMD nozzle section produced with 17-4PH type ASI 630 hard tool steel in Figure 11c presented a network of ferrite (dark etching islands) and dark spots, possibly precipitated carbide particles or porosity, in an austenitic matrix. The main difference with MFDM is that the ferritic structure is more consistent, resulting in higher hardness values. A porosity of 1.452% and average size of 104 µm was measured, and no significant defects were found (Table 5). 

The diverse microstructures result from the different material compositions, binder extractions, and bonding during sintering [25]. None of the technologies studied showed detrimental metallurgical defects, and the porosity level was acceptable. 

### 4.5. Cost Comparison

Several authors have started sharing cost comparisons on AM parts [26]. This study provides an accurate like-to-like real comparison for a specific part that illustrates cost influence as an important decision factor.

All nozzles manufactured using AM resulted in lower costs than the original part, with savings ranging from 14% to 99% (Table 6). However, only steel alloys ASI 316L and 17-4PH type 630 have sufficient strength for this application. The most economical process in machine investment and variable costs was MFDM, producing nozzles at 95% lower cost than the original parts. The most expensive process was PBF, with a 13.49% cost reduction, where variable costs and machine investment are the highest. BMD achieved savings of 63% compared to the original part. An important benefit from a operational point of view for PBF and BMD is that the entire process is controlled in-house.

## 5. Discussion

In principle, even though all three technologies are suitable for producing nozzles printed with steel or even harder materials that can withstand high pressures, Table 7 highlights several issues that need to be considered when using metal AM.

First, the PBF nozzles showed a cohesive structure and a smooth external surface waviness that would normally not affect functionality, except that external cylindrical accuracy is needed for assembly processes. In this case, external machining post-processing would be necessary. Nevertheless, further studies with PBF using steel will be required to assess if this external surface waviness is still present. With the tests conducted, parts manufactured in aluminum did not meet functional requirements, confirming the need for harder materials. Parts produced with the PBF process in steel for similar applications have provided mechanical properties and quality characteristics at least similar to MFDM and BMD parts. Producing these parts in PBF and steel would result in higher costs than original parts produced with traditional manufacturing methods and was not considered necessary to add more value to this study. An important consideration is that fixed and variable costs when manufacturing with this technology are high. Finally, handling powder metal is dangerous and requires special facility installations to guarantee minimum safety conditions. 

Second, MFDM was the most cost-efficient process, with 95% savings, and no new equipment investment was required. However, it showed significant internal material cohesion issues in several areas resulting from the lack of adherence after printing and sintering. Additionally, lead times were extremely long due to the logistics of sending parts for sintering externally. Furthermore, manual calculations were necessary to design the filament-printed green part, resulting in potential dimensional errors, inaccuracies, and model rework. Finally, during this study, the hardness measurements were significantly lower than the values specified by the vendor supplier, which may result from deficient filament adhesion during sintering by the supplier. 

Lastly, the BMD process provided parts with the highest hardness of the three and a more robust microstructure. The slicing software included a logic to produce an internal lattice structure which allows for better binder extraction and results in lower weight. However, mechanical properties such as yield strength and hardness are affected by this internal lattice and should be considered for each application. Furthermore, the parts showed several defects in the threaded and internal surface areas that did not affect functionality. Finally, from an operational point of view, there were significant furnace reliability issues due to this innovative technology.

General comments on the three technologies include the hardness measuring method, porosity defects, and recommendations for thread forming. First, Vickers HV-10 was the adequate hardness measuring method able to cover the whole range of parts, even with parts with an internal lattice, where an HRc hardness head would break through the part. Also, porosity defects found during the optical examination were acceptable and not critical, providing a structure functionally comparable to the parts made with traditional subtractive machining processes. Additionally, thread forming with AM was not accurate enough in any of the three technologies and had collateral negative form and defect creation implications. Manual thread cleaning is always necessary. Therefore, this study suggests that the most practical way to produce AM parts with threads is to print an oversized cylindrical shape and then machine the thread with a Computer Numerical Control (CNC) machine or manually with a threading tool.

Considering all, for small, precise, and complex parts, metal AM technology is highly competitive, achieving significant savings and design flexibility. BDM is a good compromise between PBF and MFDM because of the resulting quality, relatively low production lead times, and in-house control of the entire process. However, depending on the application and industry, PBF is best suited for more complex and precise parts and MFDM is best suited for less critical components where lead time is not critical.

Previous studies on AM applications in the automotive industry [4,5,6,27] have already shown the tremendous potential of this technology. Additionally, recent software development has brought numerous applications to the market to make 3D model designs easier and quicker to speed up the launch of new products [8]. However, very little work has been carried out to compare various metal AM technologies in real-world applications. This paper provides a detailed study on AM manufacturing nozzles using the most competitive AM technologies to further support the decision-making model for AM equipment investment in the automotive and other manufacturing industries.

Further research should be conducted using the other two metal AM technologies. First, DED to support sustainability objectives [10] and in combination with subtractive processes [28] for larger parts with larger tolerances. MBJ [15] is used for larger-scale AM production of parts with a broad array of parts characteristics and materials in an industrial environment without the important safety consideration from PBF (Figure 1). Furthermore, additional steel materials should be included to understand each application’s suitability. For this more extensive comparison, parts with other requirements need to be considered to cope with process limitations from any family in particular (for example, DED is limited for small and precision parts).

## 6. Conclusions

Metal AM is an excellent manufacturing technology for small, precise parts such as automotive sealant nozzles. In the Automotive Industry, MFDM is a good solution for initial trials on metal AM with no investment and costs, achieving yield strength of 680 MPa, hardness of 128 HV, acceptable porosity, and savings of over 90%. However, material detachment and hardness deviations to specified values must be considered. 

Once the first trials have been successful, BMD comes into play for small to medium-sized and complex parts without making significant investments and facility modifications. Parts achieve yield strength of 724 MPa, hardness of 307 HV10, acceptable porosity, excellent dimensional quality, and cost savings of 63%. Yield strength is impacted by the internal lattice structure incorporated by the slicing software Live Suite^TM^ (Table 2). Potential defects from this software, such as those found in the threaded area (Figure 7c), must be considered. Also, managing production with this equipment requires highly skilled personnel, mainly with the furnace.

Automotive companies should consider investing in PBF facilities to manufacture small, complex, high-volume parts that cannot be manufactured with either MFDM or BMD. Even though parts produced aluminum provided excellent finish quality, cost savings made it uncompetitive with the other two technologies. Also, PBF equipment operation requires substantial investment in special facilities to conform to safety regulations and qualified personnel. Therefore, this paper suggests installing PBF printers only in well-conditioned central labs and not on the shop floor where other products are being produced. 

Furthermore, MBJ and DED should be considered valid alternatives for larger quantities or bigger parts. DED combined with subtractive machining is a good solution for large metal parts manufacturing and repair.

This paper enhances the understanding of the metal AM equipment purchase decision tree with valuable information on quality and cost implications. With this information, the automotive industry will increase its competitive position regarding cost efficiency, just-in-time availability of parts, and sustainability. With these technologies, product development time, equipment maintenance, spare parts inventory, and logistics flow will positively impact the launch of innovative vehicles for society.

Finally, this research provides additional knowledge to the industry where cost, quality, and delivery time are fundamental. The content of this paper increases the know-how on metal AM applied to real-world applications.

## Figures and Tables

**Table 1:** Metal AM technologies families used in the automotive industry [16].

	Name	Technology
1	PBF	Powder Bed Fusion
2	DED	Direct Energy Deposition
3	MFDM	Metal Filament Deposition Modeling
4	BMD	Bound Metal Deposition
5	MBJ	Metal Binder Jetting

**Table 2:** Equipment, software, and parameters used in manufacturing the nozzle samples.

	PBF	MFDM	BMD
Equipment	PRIMA Print Genius	Ultimaker S3	DM Studio 2
Software	EP-HATCH 1.4.7	Ultimaker Cura 5.2.1	Live Studio^TM^ 3.3.2
Layer Thickness (mm)	0.03	0.1	0.1
Layer Thickness of the First Layer (mm)		0.15	
Line Width (mm)		0.6	0.9
Hatch Distance (mm)	0.18		
Hatch Angle (°)	12-79-146-213-280-347		
Infill (%)		35	
Outer/Inner Printing Power (W)	270		
In Skin Printing Power (W)	370		
Support Printing Power (W)	220		
Printing Temperature (°C)		245	
Base Plate Printing Temperature (°C)		100	
Material Flow (%)		104	
Outer/Inner Printing Speed (mm/s)	1200	25	15
In Skin Printing Speed (mm/s)	1300		
Support Printing Speed (mm/s)	2600		
Green Part Mass (g)			53.35
Finished Part Mass (g)			24

**Table 3:** Material composition of the three samples.

Material (% in Weight)	PBF	MFDM	BMD
Name/ Description	Aluminum EN AW 4343	Stainless Steel ASI 316L	Hard Tool Steel 17-4PH type ASI 630
Al	89.9		
Fe		68.4	76.8
Si	10.1	0.6	0.8
Cr		1.6	13.9
Ni		11.1	5.3
Mo		2.1	
Mn		1.1	0.4
Cu			2.8

**Table 4:** Measured yield strength and hardness.

Technology/Material	Yield Strength (MPa)	Hardness (HV10)
PBF/Al	232	109
MFDM/316L	680	128
BMD/17-4PH	724	307

**Table 5:** Porosity by technology measured with Fiji image processing software.

Technology	Material	% Porosity	Average Porosity Particle Size (µm)
PBF	EN AW 4343	1.026	8.398
MFDM	ASI 316L	0.414	2.655
BMD	17-4PH type ASI 630	1.452	103.841

**Table 6:** Summary of cost savings and functionality comparison.

Technology	Equipment	Material	%Savings (1)	Function
PBF	PRIMA Print Genius	Aluminum	13.59%	NOK
MFDM	Ultimaker	316L	94.98%	OK
BMD	DesktopMetal	17-4PH	62.84%	OK

(1) % savings calculated by comparing the original purchased part to the AM manufactured part, calculated with 3YourMind software. The cost of 3D-printed parts includes materials, labor, and overheads.

**Table 7:** Summary of issues found in the 3 trialed technologies on the sealant nozzle.

Technology	Material	Function (OK/NOK)	Issues
PBF	Aluminum	NOK	Aluminum is not sufficiently resistant High fixed and variable costs Safety concerns when handling metal powder
MFDM	ASI 316 Steel	OK	A lack of material cohesion in several areas Long lead times and potential handling damage Hardness values significantly lower than specified by the supplier
BMD	ASI 630 17-4 Hard Steel	OK	Internal lattice impact on yield resistance Potential internal defects resulting from slicing software Furnace reliability issues

## Data Availability

The original contributions presented in the study are included in the article, further inquiries can be directed to the corresponding author.
